# Evaluation of the antitumor effects of c-Myc-Max heterodimerization inhibitor 100258-F4 in ovarian cancer cells

**DOI:** 10.1186/s12967-014-0226-x

**Published:** 2014-08-21

**Authors:** Jiandong Wang, Xiaoli Ma, Hannah M Jones, Leo Li-Ying Chan, Fang Song, Weiyuan Zhang, Victoria L Bae-Jump, Chunxiao Zhou

**Affiliations:** Department of Gynecological Oncology, Beijing Obstetrics and Gynecology Hospital, Capital Medical University, Beijing, China; Department of Obstetrics and Gynecology, Division of Gynecological Oncology, University of North Carolina, Chapel Hill, NC USA; Department of Technology R&D, Nexcelom Bioscience LLC, Lawrence, MA 01843 USA

**Keywords:** Ovarian cancer, c-Myc, 10058-F4, Therapeutics, Primary cell culture

## Abstract

Epithelial ovarian carcinoma is the most lethal gynecological cancer due to its silent onset and recurrence with resistance to chemotherapy. Overexpression of oncogene c-Myc is one of the most frequently encountered events present in ovarian carcinoma. Disrupting the function of c-Myc and its downstream target genes is a promising strategy for cancer therapy. Our objective was to evaluate the potential effects of small-molecule c-Myc inhibitor, 10058-F4, on ovarian carcinoma cells and the underlying mechanisms by which 10058-F4 exerts its actions. Using MTT assay, colony formation, flow cytometry and Annexin V FITC assays, we found that 10058-F4 significantly inhibited cell proliferation of both SKOV3 and Hey ovarian cancer cells in a dose dependent manner through induction of apoptosis and cell cycle G1 arrest. Treatment with 10058-F4 reduced cellular ATP production and ROS levels in SKOV3 and Hey cells. Consistently, primary cultures of ovarian cancer treated with 10058-F4 showed induction of caspase-3 activity and inhibition of cell proliferation in 15 of 18 cases. The response to 10058-F4 was independent the level of c-Myc protein over-expression in primary cultures of ovarian carcinoma. These novel findings suggest that the growth of ovarian cancer cells is dependent upon c-MYC activity and that targeting c-Myc-Max heterodimerization could be a potential therapeutic strategy for ovarian cancer.

## Background

Among gynecologic cancers worldwide, epithelial ovarian carcinoma is the leading cause of death and the fifth most frequent cause of cancer related death across all cancers in women in the United States. Because ovarian carcinoma presents nonspecific symptoms and is often asymptomatic until late stages, the majority of patients are not diagnosed with ovarian carcinoma until they suffer from advanced stages of disease development [1,2]. Platinum/taxane chemotherapy and cytoreductive surgery have proven effective as primary treatments in patients with advanced stage ovarian carcinoma, with a positive initial response in approximately 75-80% of patients. However, most patients relapse with lethal, chemo-resistant ovarian carcinoma [3]. Rapid relapse and the development of drug resistance are the major challenges in ovarian cancer treatment that mandate the development of new adjuvant therapy for epithelial ovarian cancer.

Genetic alterations and deregulation of oncogene and tumor suppressor gene expressions are known to correlate with and promote the carcinogenesis of ovarian carcinoma. Deregulation of the expression of oncogene c-Myc is one of the most frequently encountered events present in epithelial ovarian carcinoma [4]. Myc proteins are key regulators of cell proliferation, apoptosis, and differentiation and are thus active across multiple cellular pathways [5]. Recent studies have provided strong evidence that c-Myc proteins combine with Max, a common Myc partner protein, to form heterodimers that can both bind to DNA and induce transactivation. The transcriptionally active c-Myc-Max dimer promotes proliferation, cell adhesion, apoptosis, and angiogenesis in cancer cells through its control on the transcription of Myc target genes [5,6]. The concurrent disruption of c-Myc-Max heterotetramerization interferes with the function/expression of all subsequent downstream target genes, suggesting that the c-Myc-Max interaction is a promising molecular target for cancer therapeutics. Small-molecule c-Myc inhibitor, 10058-F4, is a cell-permeable thiazolidinone that specifically disrupts the formation and function of the c-Myc-Max heterodimer and prevents transactivation of c-Myc target genes [7]. 10058-F4 exhibits potent anticancer activities towards liver, prostate, kidney, neuroblastoma, multiple myeloma, and lymphoma cells [8-13]. However, there is no evidence regarding the effect of 10058-F4 on ovarian carcinoma in vitro or in vivo. Understanding the molecular mechanisms of 10058-F4 in ovarian carcinoma cells may facilitate the development of improved therapeutic strategies for ovarian carcinoma.

In the present study, we aimed to elucidate the specific role of 10058-F4 in ovarian cancer cell growth. We used 10058-F4 to inhibit the c-Myc-Max interaction in two ovarian cancer cell lines expressing c-Myc in order to determine if the inhibition of c-Myc-Max subsequently inhibits cellular proliferation. This study provides cellular and molecular evidence for the impact of 10058-F4 on ovarian carcinoma cells through its inhibition of cell proliferation, and the induction of apoptosis and cell cycle arrest, and it offers the targeting of the c-Myc-Max interaction as a potential and viable strategy in ovarian cancer chemotherapy.

## Materials and methods

### Materials and reagents

10058-F4 (F4, Sigma, St. Louis, MO, USA) was dissolved in dimethylsulfoxide (DMSO) according the manufacturer’s instructions and further diluted to indicated concentrations in culture medium before use. SKOV3 cells were maintained in DMEM RPMI-1640 culture medium supplemented with 10% fetal calf serum (FCS), 100 U/mL penicillin, and 100 mg/mL streptomycin. Hey cells were cultured in RPMI-1640 medium with 5% FBS. Fetal bovine serum (FBS) was from Invitrogen (Carlsbad, CA, USA). The Annexin V-FITC Apoptosis Detection kit and Caspase3 Activity Assay kit were purchased from Biovision (Mountain View, CA, USA). All antibodies were purchased from Cell Signaling (Boston, MA, USA). All other materials were obtained from Life Technologies or from Sigma-Aldrich.

### MTT assays

Cells (4 × 10^3^) were seeded in 96-well microplates treated with 10058-F4 as indicated. At 72 hours after incubation, 10 μL/well of 5 mg/mL 3-(4, 5-dimethylthiazol-2-yl)-2, 5-diphenyltetrazolium bromide (MTT) solution was added to the microplates. Two hours after MTT treatment, the medium was removed, and formazan crystals were dissolved by adding 100 μL dimethylsulfoxide (DMSO) per well. Cell viability was analyzed by measuring the absorbance at 575 nm using spectrometer plate reader (Tecan, Morrisville, NC). The number of viable cells was assessed by the percentage of absorbance of treated cells relative to that of solvent controls.

### Colony formation assays

Colony formation assays were performed as described [14]. Briefly, Hey and SKOV3 cells growing in log phase were seeded (3000 cells/well in a 6-well plate) in complete regular growth medium. Cells were allowed to adhere for 24 hours, and medium was replaced with fresh complete regular growth medium containing the indicated concentrations of 10058-F4. Cells were cultured at 37°C for 10 days, with medium changes every third or fourth day. Cells were stained with 0.5% crystal violet, and colonies were counted under microscope.

### Cell cycle analysis using image cytometry

Hey and SKOV3 cells were treated with 10058-F4 for 24 hours, and cells were harvested and fixed with 70% (w/v) ice cold ethanol at 4°C for 1 hour. Fixed cells were washed twice with phosphate-buffered saline (PBS) and stained with PBS containing 50 μg/mL propidium iodide (PI) and 100 μg/mL RibonucleaseA (RNase A). Following a 30 minute incubation in darkness at 37°C, the number of cells in each cell cycle stage was analyzed using the Cellometer Vision CBA image cytometer (Nexcelom Bioscience, Lawrence, MA, USA)and FCS Express 4 (De Novo Software, Los Angeles, CA, USA). The fluorescence data was captured using FL1 with filter combination VB-660-502 (EX: 540 nm, EM: 660 nm).

### Image cytometric analysis of apoptosis

The annexin V/propidium iodide assay was performed according to the manufacturer’s recommendation. Briefly, SKOV3 and Hey cells were plated into 6-well plates and incubated for 24 hours with 10058-F4 at indicated doses. The cells were harvested, rinsed with cold PBS, re-suspended in 40 ul of binding buffer and then add 5ul FITC conjugated Annexin-V and 5ul 100 ug/ml PI and incubated for 15 min. at room temperature in the dark. A total of at least 2000 cells were collected and analyzed by image cytometry (Nexcelom Bioscience). We used FCS Express 4 software on the collected data to perform the image analysis. The fluorescence data was captured using VB-535-402 for annexin V-FITC (EX: 470 nm, EM: 535 nm) and VB-660-502 EX: 540 nm, EM: 660 nm for PI.

### ATP determination assay

The ATP concentration was assessed quantitatively using the CellTiter-Glo Luminescent cell viability assay purchased from Promega Corp. (Madison, WI, USA) according to the manufacturer’s instructions. Exponentially growing SKOV3 and Hey cells were seeded at a density of 4 × 10^3^ cells per well in white opaque 96-well plates in 100 ul of fresh culture medium containing DMEM without phenol red for 24 hours at 37°C in 5% CO_2_. Different concentrations of 10058-F4 were added to the culture medium and SKOV3 and Hey cells were incubated at 37°C for 24 hours. Then, 100 ul CellTiter-Glo Reagent was added into each well. Finally, the luminescence was read with a microplate reader with an integration time of 1 second per well. The experiments were independently performed three times and each 10058-F4 treatment was performed in triplicate.

### Reactive oxygen species (ROS) assay

ROS generation was assessed using an ROS-sensitive fluorescence indicator DCFH-DA and MitoSox red. To determine intracellular and mitochondria ROS scavenging activity, SKOV3 and Hey cells (1.0 × 10^4^ cells/well) were seeded in black 96-well plates. After 24 hours, the cells were treated with 10058-F4 for 8 hours to induce ROS generation. After the cells were incubated with DCFH-DA (20 μM) or MitoSox (10 um) for 30 min., the fluorescence intensity was measured at an excitation wavelength of 485 nm and an emission wavelength of 530 nm for DCFH-DA and emission 590 nm for MitoSox using a Tecan spectrometer plate reader.

### Caspase-3 activity assay

Caspase-3 activity was detected by using caspase-3 activity assay kit (Biovision). This assay is a Fluorometric assay which measures caspase-3 activities. The level of caspase-3 activity is measured by detection of cleavage of substrate DEVD-AFC. Briefly, Primary culture cells (1.5 × 10^5^ cells/well) or ovarian cancer cells (2.5 × 10^5^ cells/well) were seeded in 12 well plates or 6 well plates and incubated for 24 hours at 37°C. The cells were treated with 10058-F4 at different concentration for 24 hours and then lysated with lysis buffer. 50 ug cell lysates was used for measuring caspase-3 activity following manufacture instructions. The samples were reading in 96 well plates at a fluorometric plate reader.

### Western blot analysis

Total proteins from SKOV3 and Hey cells were extracted in lysis buffer (Thermo Fisher Scientific, Rockford, IL) and quantified using the BCA method. Thirty micrograms of protein were separated by 12% SDS–PAGE gel. After transfer in cold room, the polyvinylidene fluoride (PVDF) membranes (Millipore, Billerica, MA, USA) were incubated overnight at 4°C with the following antibodies: beta-actin(1:3000; Santa Cruz Biotechnology, Santa Cruz, CA), cyclin D1(1:1000), CDK4(1:1000), CDK6(1:1000), p27 (1:1000) and p21(1:1000) (Cell Signaling, Boston, MA, USA). After incubation with peroxidase-coupled anti-mouse IgG and anti-rabbit IgG (Santa Cruz Biotechnology) at 37°C for 1 hour, bound proteins were visualized using ECL (Thermo Fisher Scientific) and detected using x films. The relative protein levels were calculated based on β-actin as the loading control.

### Primary cell culture of ovarian carcinoma

Primary ovarian carcinoma samples were collected in the operating room of the Department of Gynecologic Oncology, Beijing Obstetrics and Gynecology Hospital, Capital Medical University. Beijing, China. A specific written informed consent was obtained from patients, and the study was approved by the Institutional Ethics Committee of Beijing Obstetrics and Gynecology Hospital, Capital Medical University, Beijing, China. In brief, the freshly obtained ovarian carcinoma tissues were washed three times in sterile, cold Hank’s Buffered Salt Solution (HBSS) to remove blood and secretions, and then gently minced by scissors in DMEM/F12 medium containing 10% fetal calf serum (FCS). These tumor cells were then incubated for 1–2 hours at 37°C in 10 ml DMEM/F12 supplemented with 0.5% collagenase IA, 0.1% DNase, 100 U/ml penicillin and streptomycin with occasional shaking. After two centrifugations with PBS wash, the tumor cells were suspended in DMEM/F12 medium and then diluted to 1 × 10^5^ cells/ml. Aliquots (100 μl) of tumor cell suspension were plated into 96 well tissue culture plates resulting in approximately 2 × 10^4^ cells per well. The cells were incubated at 37°C for 24 hours in a 5% CO2 incubator, and then were treated with various concentrations of 10058-F4 or DMSO. Cell proliferation was assessed using an MTT assay.

## Results

### 10058-F4 inhibits cell proliferation and colony formation

**Figure 1:**
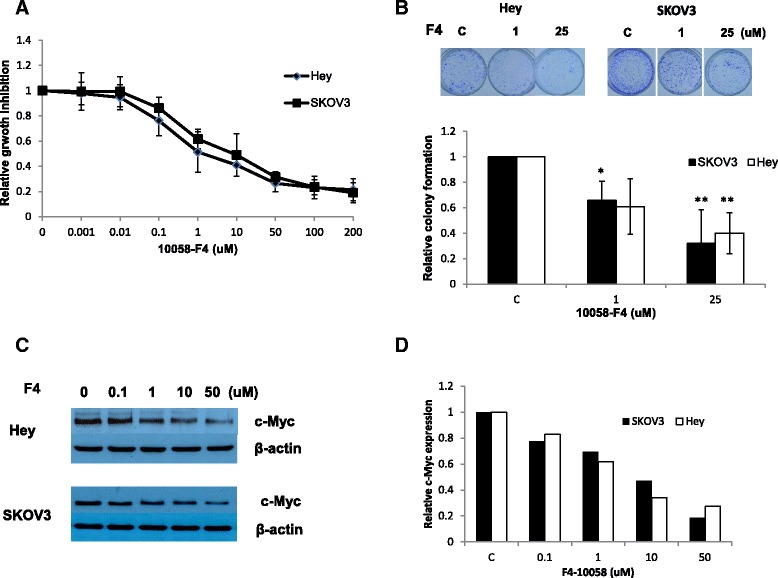
**10058-F4 inhibits cell proliferation in ovarian cancer cells.** SKOV3 and Hey cells were cultured for 24 hours and treated with F4-10058 as indicated doses in 96 well plates for 72 hours. Cell proliferation was assessed with MTT assay **(A)**. 10058-F4 effect on long term growth in ovarian cancer cells was assessed through colony-forming assay **(B)**. Western blotting results indicated F4-10058 inhibited c-Myc protein expression in a dose dependent manner after 36 hours treatment **(C and D)**. *p < 0.05 and **p < 0.01, two-sided student t test.

To investigate the potential inhibition of cell growth by 10058-F4 in ovarian cancer, we first examined the effect of 10058-F4 on cell proliferation and clonogenic survival in SKOV3 and Hey cells. 10058-F4 induced anti-proliferative activities in SKOV3 and Hey cells, inhibiting growth in both cell lines in a concentration-dependent manner (Figure 1A). The IC50 values of 10058-F4 were 4.4 μmol/L for SKOV3 and 3.2 μmol/L for Hey, respectively. We next questioned whether 10058-F4 had an effect on the colonization ability of the cell lines, considering that in vitro colony formation assays are excellent indicators of long term tumor cell survival and enable predictions of the long term antitumor effects of drugs [15]. We observed that clonogenicity of both cancer lines was reduced in a concentration-dependent manner after exposure to 10058-F4 for 10 days (Figure 1B). Together, these results demonstrate suppressive effects of 10058-F4 on the proliferation of ovarian cancer cells.

Finally, we used western blotting to evaluate the effects of 10058-F4 treatment on c-Myc protein expression. 10058-F4 is known specifically to inhibit the c-Myc-Max interaction, decrease c-Myc protein expression, and prevent transactivation of c-Myc target genes [9,10]. As we expected, 10058-F4 treatment for 36 hours induced a marked decrease in the protein expression of c-Myc in a dose-dependent manner in both cell lines when compared to the control group (Figure1C and D).

### 10058-F4 induces cell cycle arrest

**Figure 2:**
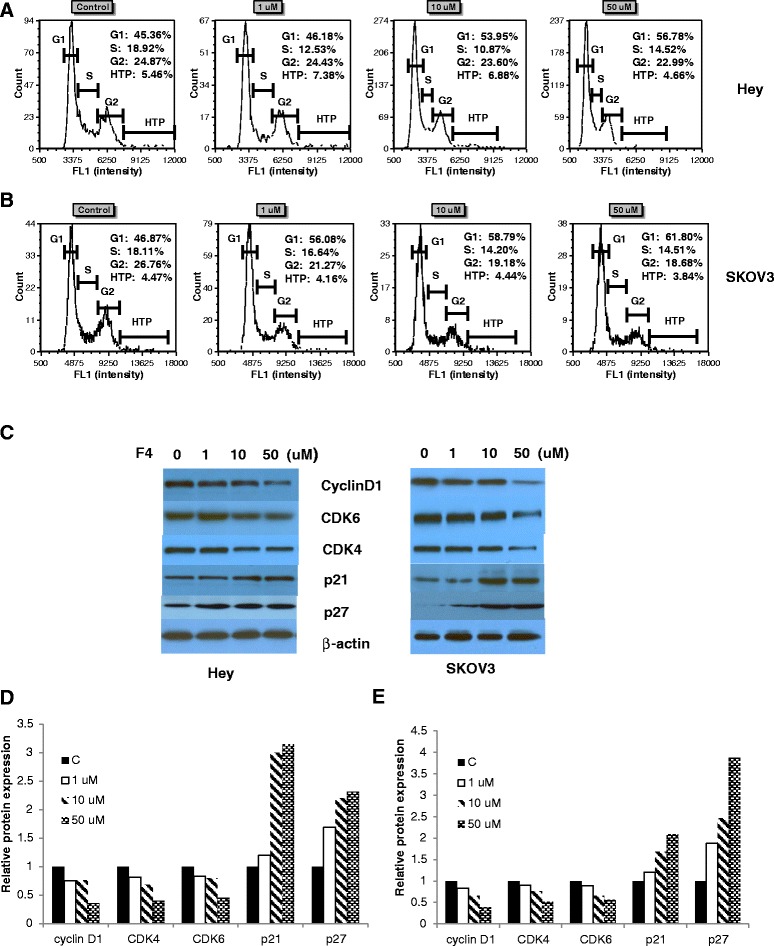
**10058-F4 induced cell cycle G1 arrest in ovarian cancer cells.** SKOV3 and Hey cells were treated with 10058-F4 at the indicated dose for 48 hours and then analyzed for cell cycle distributions by Cellometer **(A and B)**. Western blotting analysis of cyclinD1, CDK4, CDK6, p21, p27 and β-actin expression in ovarian cancer cells treated with 10058-F4 for 24 hours **(C)**. The alternations of cyclinD1, CDK4, CDK6, p21 and p27 were summarized in **D** and **E**. *p < 0.05 and **p < 0.01, two-sided student t test.

To examine the mechanism responsible for 10058-F4-mediated inhibition of cell growth, Hey and SKOV3 cells were treated with the indicated concentrations (0–50 uM) of 10058-F4 for 48 hours. The cell cycle was determined by image cytometry. Our data (Figure 2A and B) show that, consistent with the growth suppression observed in the MTT and colony formation assays, 10058-F4 treatment led to an increased accumulation of cells in the G1 phase and a concomitant decrease in the number of S phase cells in both cell lines when compared with the control groups. It has been reported that Myc overexpression enhances endoreduplication in cancer cells [16]. We next analyzed the effects of 10058-F4 on endoreduplication in both cell lines by image cytometry. However, treatment cells with 10058-F4 did not affect the percentage of hypertetraploid phase (HTP) in SKOV3 and Hey cells.

To further understand the molecular events underlying the observed G1 arrest, we next examined the effects of 10058-F4 on key regulatory molecules including Cyclins D1, CDK4 and CDK6 which co-operate to promote the transition from G1 to S phase. Expression of CDK4 and CDK6 and Cyclin D1 decreased in 10058-F4-treated SKOV3 and Hey cells. Because c-Myc gene antagonizes the activity of cell cycle inhibitors as p21 and p27 through different mechanisms, both proteins were analyzed by Western blotting in response to 10058-F4 after 24 hours of treatment (Figure 2C-E). The levels of p21 and p27 protein expression were increased in a dose dependent manner. These results indicated that 10058-F4 causes growth inhibition associated with induction of G1 phase arrest.

### 10058-F4 induces apoptosis

**Figure 3:**
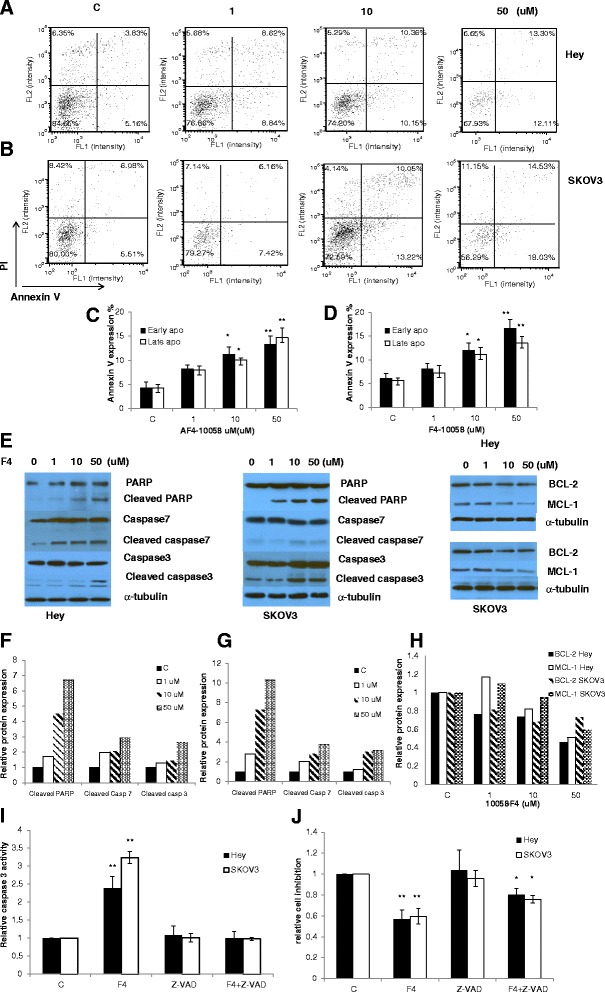
**10058-F4 induced apoptosis in ovarian cancer cells.** SKOV3 and Hey cells were treated with 10058-F4 at the indicated doses for 24 hours and then analyzed for AnnexinV and PI staining by Cellometer **(A and B)**. The measurement of early and late apoptosis was shown in **C** (Hey) and **D** (SKOV3) at two independent experiments. Hey and SKOV3 cells treated with 10058-F4 for 16 hours were analyzed by Western blotting using PARP, capspase3, caspase7, BCL-2, MCL-1 and β-actin antibodies **(E)**. The BCl-2 and MaCL-1 were analyzed by Western blotting after 16 hours treatment with 10058-F4. 10058-F4 increased cleaved PARP, caspase3 and caspase7 protein expression **(F and G)** and decreased BCL-2 and MCL-1 protein expression **(H)**. Caspase-3 activity **(I)** and cell proliferation **(J)** were determined by ELISA and MTT assay after the both cells were treated with Z-VAD-FMK, 10058-F4 or combination for 24 or 48 hours. *p < 0.05 and **p < 0.01, two-sided student t test.

To verify that 10058-F4 treatment inhibits cell proliferation by inducing apoptosis, we investigated apoptotic cells by applying an Annexin-V and PI double staining assay after a 24 hours treatment. As shown in Figure 3A-D, the percentage of SKOV3 and Hey cells undergoing early and late apoptosis significantly increased in a dose-dependent manner when compared to the control (p < 0.05). We next determined whether mitochondrial apoptosis pathway which leads to caspase activation and induces cell death was involved in 10058-F4-induced apoptosis in ovarian cancer cells. We treated both cells with the indicated concentration of 10058-F4 for 16 hours, and cleaved caspase-3, caspase-7, and PARP proteins were determined by Western blotting using antibodies that specifically detect the cleave forms of the caspases. We observed a dose-dependent increase in expression of cleaved caspase proteins in both cell lines in response to 10058-F4. In addition, we found 10058-F4 reduced BCL-2 and MCL-1 protein expression in a dose dependent manner after treatment of 10058-F4 for 24 hours (Figure 3E-H).

To further verify the direct role of apoptosis in 10058-F4-treated ovarian cancer cells, we used pan-caspase inhibitor (Z-VAD-FMK) to block caspase activity in both cell lines and determined whether the cell proliferation inhibition and caspase-3 activity were changed after 10058-F4 treatment. Pretreatment with Z-VAD-FMK resulted in total blocking of 10058-F4 induced caspase-3 activity and a significant decrease in the inhibition of 10058-F4-mediated proliferation in both cells (Figure 3I and J), suggesting that inducing mitochondrial apoptosis may be a major mechanism to inhibit cell proliferation in 10058-F4 treated ovarian cancer cells.

### 10058-F4 decreases Reactive Oxygen Species (ROS) and ATP generation

**Figure 4:**
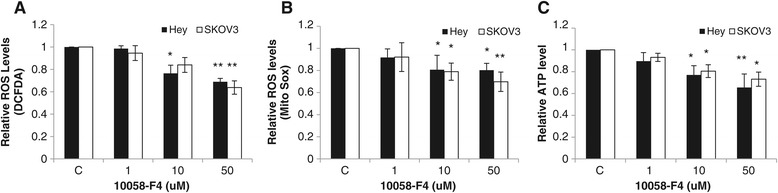
**Effect of 10058-F4 on oxidative stress and ATP production in ovarian cancer cells.** Hey and SKOV3 cells were treated with F4-10058 at the indicated doses in 96 well plates for 24 hours. Reactive oxygen species (ROS) and ATP production were analyzed by plate reader. Data are presented as the mean of three independent experiments **(A, B and C)**. *p < 0.05 and **p < 0.01, two-sided student t test.

Reactive Oxygen Species (ROS) are required for ovarian cancer cell growth [17]. Myc is known to induce ROS accumulation. The increase in ROS levels may result in significant damage to cellular structures and lead to fatal lesions in cells that contribute to the inhibition of cell growth. To evaluate whether targeting c-Myc-Max interaction by 10058-F4 influences the generation of ROS, we assessed intracellular ROS production with DCFDA in both cell lines treated with different concentrations of 10058-F4 for 24 hours. As shown in Figure 4A, intracellular ROS concentration gradually decreased with increasing concentrations of 10058-F4. Because mitochondrial respiration serves as a principal source of ROS in most cells, we next examined whether 10058-F4 affected mitochondria ROS production with MitoSox red, which is a new redox-sensitive dye that is targeted to mitochondria. Treatment with 10058-F4 for 24 hours in both ovarian cancer cells resulted in a significant decrease in mitochondria ROS levels (Figure 4B), suggesting 10058-F4 may inhibit the mitochondrial electron transport chain through the reduction of the c-Myc and Max interaction (21). Under the same conditions, we also found a reduced level of cellular ATP production in a dose-dependent manner (10–50 uM) in each cell line after a 24 hour treatment, showing reduced levels of ATP production of around 40% and 30% in Hey and SKOV3 cells, respectively (Figure 4C). Thus 10058-F4 reduced ROS generation and inhibited cellular ATP production, implicating the relevance of ROS presence and ATP reduction in the cytotoxicity of 10058-F4 in ovarian carcinoma cells. These results suggest that a reduction of cellular ATP and inhibition of mitochondrial respiration might be involved in cytotoxicity of 10058-F4 in ovarian carcinoma cells.

### Cytotoxicity of 10058-F4 in primary cultures of ovarian carcinoma

**Figure 5:**
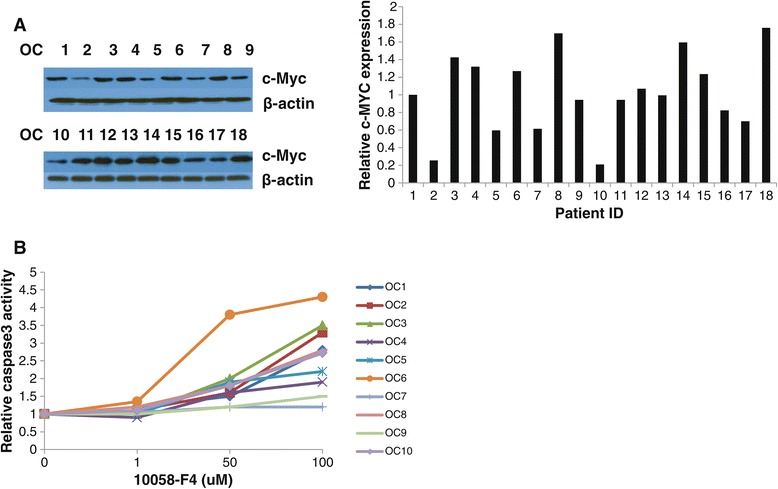
**c-MYC protein expression in primary culture cells of ovarian cancer.** Extract protein from untreated human primary culture cells of ovarian cancer. c-Myc protein expression was determined by Western Blotting **(A)**. Caspase-3 activity was determined in ten primary cell cultures after a 72 hours treatment with 10058-F4 **(B)**.

To further determine the clinical relevance of 10058-F4, we examined the effect of 10058-F4 in primary cultures of ovarian cancer. These tissue samples were obtained from patients undergoing surgery for primary epithelial ovarian cancer. After 72 hours of treatment, 15 of the 18 individual patient cultures responded to the 10058-F4 treatment, showing a significant level of cytotoxicity and inhibition of cell growth. 12 of the 15 sensitive culture samples exhibited significantly reduced cell proliferation under 10058-F4 treatment, with wide ranges of IC 50 values from 16–100 μM (Table 1). In order to determine the relationship between the level of c-Myc protein expression and sensitivity to 10058-F4 in each case, we detected c-Myc protein expression using Western blotting in 18 untreated primary cell cultures. The western blotting revealed different levels of over-expressed c-Myc in the 18 primary cultures, and an analysis of the data using a linear regression model (data not shown) showed distinct, significant, responses to 10058-F4 independent of the precise level of c-Myc protein over-expression (Figure 5A). The growth of three primary cell cultures with different expression levels of c-Myc was not affected by 10058-F4 at a dose of 1–100 uM, suggesting that a higher concentration of 10058-F4 may be needed in these three samples or that these cultures may contain an excess of stromal cells (13). To verify the apoptotic effects of 10058-F4 in the primary cultures, caspase-3 activity was determined first with ten primary cell cultures after a 72 hours treatment. There are no relationships between level of c-Myc protein expression and caspase-3 activity. No obvious changes of caspase-3 activity in 2 of the primary cell cultures were observed at concentrations of 10058-F4 up to 100 uM (Figure 5B). However, the 8 primary cultures that had achievable IC50 values showed an increase in different levels of caspase-3 activity, suggesting that the ability of 10058-F4 to inhibit cell proliferation in primary cultures may be mainly dependent on the induction of apoptosis.

**Table 1:** **Pathologic features and inhibition of cell growth by 10058-F4 in primary culture cells of ovarian carcinoma**

**Patient ID**	**AGE**	**Diagnosis**	**Stage**	**Grade**	**IC50 (uM)**	**c-MYC**
avb	65	Serous	IIIA	G1	27	Positive
OC2	72	Serous	IIIC	G3	84	Positive
OC3	48	Serous	IIA	G1	28	Positive
OC4	36	Serous	IC	G1	56	Positive
OC5	66	Serous	IIIA	G1	26	Positive
OC6	61	Mucous	IIIA	G2	>100	Positive
OC7	55	Serous	IIIC	G3	N	Positive
OC8	51	Serous	IIA	G3	62	Positive
OC9	69	Serous	IIIC	G3	>100	Positive
OC10	53	Serous	IIIA	G1	30	Positive
OC11	38	Mucous	IA	G1	>100	Positive
OC12	47	Serous	IIIC	G3	N	Positive
OC13	64	Mucous	IIIA	G1	18	Positive
OC14	66	Serous	IIIC	G3	N	Positive
OC15	62	Serous	IIIA	G2	>100	Positive
OC16	57	Serous	IIIA	G1	22	Positive
OC17	43	Serous	IIA	G3	33	Positive
OC18	51	Serous	IIIC	G3	16	Positive

18 primary culture cells of ovarian cancer were cultured in 96 well plates or 6 well plates and treated with 10058-F4 as indicated doses. Cell proliferation was assessed by MTT assay.

## Discussion

Accumulating evidence suggests that the Myc family is an excellent target for anti-cancer therapeutics due to its involvement in cell growth, metabolism, proliferation, apoptosis, and differentiation. Numerous c-Myc targeting strategies, including the inhibition of c-Myc expression or the interruption c-Myc and its downstream effects, are currently being used in experimental therapeutics for several types of cancer [5,18]. Most of these approaches continue to be hampered by technical difficulties pertaining largely to delivery and the fact that many c-Myc target genes are functionally redundant and/or cell type specific [19]. Over the last several years, several groups have developed small molecular inhibitors that interfered with the interaction between c-Myc and Max. These inhibitors restrain not only c-Myc-Max heterodimerization but also all subsequent downstream functions of c-Myc. 10058-F4 is a small molecular inhibitor that is composed of a six-member ethylbenzylidine ring and a five-member thioxothiazolidin-4-one. It specifically inhibits the dimerization of Myc and Max and prevents the transactivation and expression of the c-Myc target genes in doses up to 100 uM (13, 19). Several studies have confirmed that 10058-F4 significantly inhibited the growth of cancer cells in vitro through the induction of apoptosis and cell cycle G1 arrest [8-13,20]. A recent animal study has shown treated mice with 10058-F4 for 14 days was sufficient to delay tumor growth in a neuroblastoma transgenic mouse model and a xenograft mouse model, These data indicated that delaying tumor growth in vivo can be achieved by using a small molecule inhibitor that targets the c-Myc-Max interaction and targeting c-Myc-Max heterodimerization might be a good strategy for development of cancer therapy [20]. Although clinical development of c-Myc-Max inhibitors, including 10058-F4, is limited by their relatively low potencies [19], we needed to explore the effect of targeting the interaction between c-Myc and Max by 10058-F4 on ovarian cancer cells because little or no advancements have been made in ovarian cancer treatment over the last thirty years. The present study is the first to demonstrate that blocking the interaction between c-Myc and Max by 10058-F4 reduces the viability of ovarian carcinoma cells by reducing intracellular ATP and ROS production, downregulating c-Myc expression and inducing apoptosis and cell cycle arrest in ovarian cancer cells. Such inhibitions in ovarian cancer cells, caused by 10058-F4, are ultimately consistent with the results of other study groups [8-13,20]. Our primary result of general toxicity in mice showed 10058-F4 did not cause obvious toxic effects when injected 1008-F4 at a dose of 25 mg/kg daily for 5 days (data not shown). These results present further evidence that the c-Myc-Max signaling pathway is essential to ovarian cancer development and progression and that the c-Myc-Max interaction may be used as an effective molecular target for ovarian cancer therapy [7,19,21].

One of the most striking observations in this study is the sensitivity of the ovarian cancer cells to the anti-proliferative effects of 10058-F4 despite the variations in c-Myc protein expression across the ovarian carcinoma cell lines and primary ovarian carcinoma cells, which is consistent with Holien’s results in study of multiple myeloma [13]. These results clearly pose an interesting possibility: that the concurrent disruption of c-Myc-Max heterotetramerization might also interfere with target gene expression in other ways in various cell types [19,21]. This result could also account for the heterogeneity of primary cultures of ovarian cancer, showing small portions of stromal cells in some cases in our primary culture system, and this presence of stromal cells may affect the viability of and c-Myc function in the ovarian primary cultures.

Increased metabolic activity and glucose consumption in ovarian cancer patients have been linked to ovarian cancer aggressiveness [22-24]. Cellular ATP levels are a valuable predictor for chemo-resistance in cancer cells [25]. The c-Myc oncogene is a major controller of many aspects of cellular metabolism through the regulation of genes related to glucose metabolism, glutamine metabolism, and mitochondrial biogenesis [6]. Transgenic mice with an overexpression of c-Myc in the liver had increased glycolytic enzyme activity and production of lactic acid in the liver [26]. Inhibition of c-Myc by SiRNA or small molecular inhibitors led to the reduction of glucose uptake and activities of glucose glycolytic enzymes (HKII, LDHA), all of which ultimately reduce ATP production [27-29]. Overexpression of Myc induces nuclear encoded mitochondrial gene expression and biogenesis and increases the production of mitochondrial ROS [16]. c-Myc knockdown is associated with a reduction in ROS production and inhibition of autophagy in cancer cells [30]. In agreement with previous studies on ATP reduction through the inhibition of c-Myc, the metabolic and therapeutic stress induced by 10058-F4 led to an acute ATP depletion, which was accompanied by decreased intracellular and mitochondrial ROS and ultimately led to the inhibition of cell growth in ovarian carcinoma cells.

Assessment of chemotherapeutic drug sensitivities using primary culture cancer cells provides clinically relevant information for the optimization of the cancer patient’s treatment [31-33]. In the panel of 18 primary cell cultures of primary epithelial ovarian carcinoma, inhibition of cell proliferation was observed in 15 primary cultures after 72 hours of treatment. The level of c-Myc protein expression in untreated cells from primary cultures was not associated with sensitivity to 10058-F4 [13]. However, caspase-3 activity correlated strongly with cellular response to 10058-F4 in 10 primary cultures of ovarian cancer, suggesting that the induction of apoptosis is a major action for the small molecular inhibitors that target the interaction of c-Myc-Max. The results indicated that suppression of c-Myc through the targeting of the c-Myc-Max heterodimer has broad therapeutic applications in cancers including ovarian cancer [7,19,20].

## Conclusions

Inhibition of the Myc-Max heterodimer resulted in promising anti-tumor activity in ovarian cancer cell lines and ovarian cancer primary cultures and led to metabolic alterations. Efficient and selective inhibition of c-Myc through the targeting of c-Myc-Max interaction is thus a compelling strategy for treatment of ovarian cancer.
